# Association Between Polymorphisms rs11003125 and rs7096206 of the *MBL2* Gene and the Stages of Hepatitis B Progression in Burkina Faso: A Comparative Cross‐Sectional Study

**DOI:** 10.1002/hsr2.72454

**Published:** 2026-04-27

**Authors:** Minane Nafissa Triande, Lassina Traore, Sidnooma Véronique Zongo, Teega‐Wendé Clarisse Ouedraogo, Mousso Savadogo, Sanata Nadine Kiemde, Tegwindé Rébéca Compaore, Augustin Tozoula Bambara, Roger Arsène Sombie, Patoinewendé Denise Ilboudo, Albert Théophane Yonli, Bolni Marius Nagalo, Florencia Wendkuuni Djigma, Jacques Simpore

**Affiliations:** ^1^ Laboratoire de Biologie Moléculaire et de Génétique (LABIOGENE) Université Joseph KI‐ZERBO Ouagadougou Burkina Faso; ^2^ Centre de Recherche Biomoléculaire Pietro Annigoni (CERBA) Ouagadougou Burkina Faso; ^3^ Université Norbert Zongo – Centre Universitaire de Manga Koudougou Burkina Faso; ^4^ Unité de Formation et de Recherche en sciences de la santé Université Joseph KI‐ZERBO Ouagadougou Burkina Faso; ^5^ Université Yembila Abdoulaye TOGUYENI Fada N'Gourma Burkina Faso; ^6^ Department of Pharmacology and Physiology University of Maryland School of Medicine Baltimore Maryland USA; ^7^ Marlene and Stewart Greenebaum NCI Comprehensive Cancer Center University of Maryland School of Medicine Baltimore Maryland USA

**Keywords:** Burkina Faso, gene, HBV, MBL2, polymorphisms

## Abstract

**Background and Objectives:**

Hepatitis B virus (HBV) infection remains a major global health burden, with disease progression influenced by host genetic and immune factors, including variants in the mannose‐binding lectin 2 (MBL2) gene. This study aimed to evaluate the association between MBL2 polymorphisms and clinical outcomes of HBV infection in a cohort from Burkina Faso.

**Method:**

A total of 74 participants were recruited in 2022, including individuals with chronic hepatitis B (CHB, *N* = 12), hepatocellular carcinoma (HCC, *N* = 28), cirrhosis (*N* = 12) and resolved hepatitis B (*n* = 22). Genotyping of MBL2 promoter polymorphisms (rs11003125 and rs7096206) was performed using real‐time PCR (QuantStudio 5). Statistical analyses were conducted using SPSS v20 and Epi Info v7.5.2.0, with significance defined as *p* < 0.05 (Fisher's exact test).

**Results:**

For rs11003125, the GC heterozygous genotype predominated (77%), followed by GG (16.2%) and CC (6.7%). For rs7096206, genotype frequencies were 43.2% (CC), 28.4% (CG) and 28.4% (GG). The rs7096206 GG genotype and G allele were strongly associated with infection resolution (OR = 0.02, 95% CI: 0.0002–0.3, *p* < 0.001; and OR = 0.06, 95% CI: 0.01–0.2, *p* < 0.001, respectively), and with reduced risk of progression from cirrhosis to HCC (OR = 0.25, 95% CI: 0.09–0.7, *p* = 0.009). Additionally, the rs11003125 C allele was associated with a decreased risk of progression to HCC (OR = 0.3, 95% CI: 0.1–0.7, *p* = 0.01).

**Conclusion:**

From an exploratory perspective, the analysis of the rs11003125 and rs7096206 polymorphisms located in the promoter region of the MBL2 gene suggests their possible involvement in the progression of HBV infection. These preliminary results support the hypothesis of a potential functional role of this gene in HBV pathogenesis, while highlighting the need for further studies to confirm and clarify these associations.

**Abbreviations**
CHB: chronic hepatitis B
DNA: deoxyribonucleic acid
HBcAb: anti‐HBc antibodies
HBeAb: anti‐HBe antibodies
HBeAg: HBe antigen
HBsAb: anti‐HBs antibodies
HBsAg: HBs antigen
HBV: hepatitis B virus
HCC: hepatocellular carcinoma
HCV: hepatitis C virus
HIV: human immunodeficiency virus
*MBL2*
: mannose‐binding lectin 2
RHB: resolved hepatitis B
rs: reference of SNP
SNP: single nucleotide polymorphism

## 1 Introduction

Hepatitis B virus (HBV) infection remains a major global health challenge, causing acute and chronic liver disease with substantial morbidity and mortality [1].

Despite the availability of an effective vaccine, HBV continues to disproportionately affect low‐ and middle‐income countries, with an estimated 254 million individuals living with chronic infection, 1.2 million new infections annually, and approximately 1.1 million deaths, primarily due to cirrhosis and hepatocellular carcinoma (HCC) [2].

In sub‐Saharan Africa, where HBV is highly endemic, HCC represents a leading cause of cancer‐related mortality. HCC is the fourth leading cause of cancer mortality, with a rate of 8.2% [3].

In Burkina Faso, the burden of HBV is particularly high, with prevalence among blood donors increasing from 7.8% in 2018 [4] to 11.87% in 2021 [5].

The clinical course of HBV infection is highly heterogeneous, ranging from spontaneous viral clearance to chronic infection and progressive liver disease, including cirrhosis and HCC. In 20% of cases, chronic infection sets in and can progress to severe forms such as liver cirrhosis and HCC [6].

While viral factors contribute to disease progression, accumulating evidence indicates that host immune and genetic determinants play a central role in shaping clinical outcomes [7, 8]. Polymorphisms in immune‐related genes, including HLA alleles (HLA‐DRB1*11, HLA‐DRB1*12), cytokines such as interleukin‐10 (IL‐10) and tumor necrosis factor‐α (TNF‐α), and co‐stimulatory molecules such as CD40, have been associated with susceptibility to infection, viral persistence and disease severity across diverse populations [9, 10, 11, 12, 13]. However, these associations remain incompletely characterized in African cohorts.

Mannose‐binding lectin (MBL), a liver‐derived pattern recognition molecule, is a key component of innate immunity that mediates pathogen recognition and activates the lectin pathway of complement [14]. Beyond its role in microbial opsonization, MBL modulates inflammatory responses by regulating cytokine production, including TNF‐α, IL‐6 and IL‐1β, thereby influencing both antiviral defense and immunopathology [15].

Circulating MBL levels are largely determined by genetic variation in the MBL2 gene, located on chromosome 10, where several polymorphisms, particularly within the promoter region, affect transcriptional activity and protein expression. Among these, rs11003125 and rs7096206 have been shown to significantly influence MBL expression [16].

Variants associated with reduced MBL production may impair complement activation and viral clearance, favoring chronic infection, whereas variants linked to higher MBL expression may enhance viral control but potentially exacerbate inflammatory liver damage. Although MBL2 polymorphisms have been implicated in susceptibility and progression of infectious diseases, their role in HBV pathogenesis remains incompletely understood, particularly in African populations where both HBV burden and genetic diversity are high. No data are currently available on the contribution of MBL2 genetic variation to HBV disease progression in Burkina Faso. Here, we investigated the association between promoter polymorphisms in MBL2 (rs11003125 and rs7096206) and clinical outcomes of HBV infection in a Burkinabe cohort encompassing chronic hepatitis B (CHB), cirrhosis, HCC and resolved infection. By integrating host genetic variation with disease phenotypes, this study aims to elucidate the role of the lectin complement pathway in HBV pathogenesis and to identify potential biomarkers of disease progression in high‐burden settings.

## 2 Materials and Method

### 2.1 Study Design and Participants

This comparative cross‐sectional study was conducted in Ouagadougou, Burkina Faso, between August and December 2022. A total of 74 participants were recruited and stratified into 4 clinical groups: CHB (*n* = 12), HBV‐related cirrhosis (*n* = 12), HBV‐related HCC (*n* = 28), and resolved hepatitis B (RHB, *n* = 22). Participants with cirrhosis and HCC were recruited from the hepatogastroenterology departments of CHU Yalgado Ouédraogo and Paul VI Hospital. Individuals with CHB were enrolled at the Centre de Recherche Biomoléculaire Pietro Annigoni (CERBA), whereas participants with resolved infection were recruited from the Centre National de Transfusion Sanguine.

### 2.2 Inclusion and Exclusion Criteria

#### 2.2.1 Inclusion

Clinical classification was based on serological, radiological, and/or histological criteria. CHB was defined by persistent HBsAg positivity for > 6 months without significant liver abnormalities on ultrasound. Cirrhosis was clinically confirmed with HBV as the sole etiological factor. HCC diagnosis was based on elevated alpha‐fetoprotein (AFP), imaging (computed tomography), and/or histological confirmation, with HBV as the primary cause. Resolved infection was defined by HBsAg negativity, anti‐HBs positivity, anti‐HBc positivity, absence of HBeAg, presence of anti‐HBe, and undetectable viral load (Table 1).

**Table 1:** Study inclusion criteria.

Clinical groups	Inclusion criteria
Chronic HBV	– Confirmed HBV infection for more than 6 months – Demonstrated by HBsAg positivity – Ultrasound results showing no significant liver abnormalities
Cirrhosis	– Clinically confirmed hepatic cirrhosis – HBV being the only etiological agent
HCC	– Recruitment based on results of alpha‐fetoprotein (AFP) test, CT scan, and/or histological examination of liver – HBV as the sole etiological agent
Resolved HBV	Anyone: – HBsAg negative – HBsAb positive – HBeAg negative – HBeAb positive – HBcAb positive – With an undetectable viral load

#### 2.2.2 Non‐Inclusion Criteria

Exclusions included cases of HIV seropositivity, HCV seropositivity, individuals refusing to participate in the study, and individuals who did not provide explicit written informed consent.

### 2.3 Sample Collection and DNA Extraction

Following informed consent and structured clinical data collection, 10 mL of peripheral blood was obtained by venipuncture into ethylenediaminetetraacetic acid and dry tubes. Plasma and serum were separated by centrifugation and stored for downstream analyses. Genomic DNA was extracted using the FAVORGEN kit according to the manufacturer's protocol, including cell lysis, protein precipitation and DNA purification steps. DNA concentration and purity were assessed by spectrophotometry (BioDrop).

### 2.4 Genotyping of *MBL2* Gene Polymorphisms rs11003125 and rs7096206

Genotyping of MBL2 promoter polymorphisms (rs11003125 and rs7096206) was performed using TaqMan allelic discrimination assays on a QuantStudio 5 Real‐Time PCR system (Applied Biosystems). Probes labeled with VIC and FAM fluorophores were used to distinguish alleles. Each 25 µL reaction contained 17.5 µL nuclease‐free water, 3 µL FIREPol Probe Universal qPCR Mix [5×], 1.5 µL diluted TaqMan SNP genotyping assay (1:5 from 40× stock), and 3 µL genomic DNA. Thermal cycling conditions consisted of an initial denaturation at 95°C for 10 min, followed by 40 cycles of 95°C for 15 s and 60°C for 60 s. Genotype calls were assigned using QuantStudio software based on fluorescence signal discrimination.

We referred to two protocols for genotyping MBL2 gene polymorphisms, adapting some parameters [17, 18].

### 2.5 Statistical Analysis

Allelic and genotypic frequencies were calculated, and associations with clinical phenotypes were evaluated using SPSS v20 and Epi Info v7.2.5.0. Odds ratios (ORs) with 95% confidence intervals (CIs) were estimated using binary logistic regression for pairwise comparisons between clinical groups, with MBL2 genotypes as independent variables. Statistical significance was assessed using two‐tailed Fisher's exact tests, with *p* < 0.05 considered significant. Given the exploratory nature of the study and limited sample size, analyses were not adjusted for multiple comparisons.

### 2.6 Ethical Approval

The study was approved by the Ethics Committee for Health Research of Burkina Faso (Reference No. 2022‐02‐027). All participants provided written informed consent prior to enrollment. Data confidentiality was ensured through secure, password‐protected storage systems.

## 3 Results

A total of 74 participants were included and stratified according to clinical stage of HBV infection: CHB (16.2%: 12/74), cirrhosis (16.2%: 12/74), HCC (37.8%: 28/74), and RHB (29.7%: 22/74). The cohort comprised 46 males (62.2%: 46/74) and 28 females (37.8%: 28/74), corresponding to a sex ratio of 1.64 (46/28).

**Figure 1:**
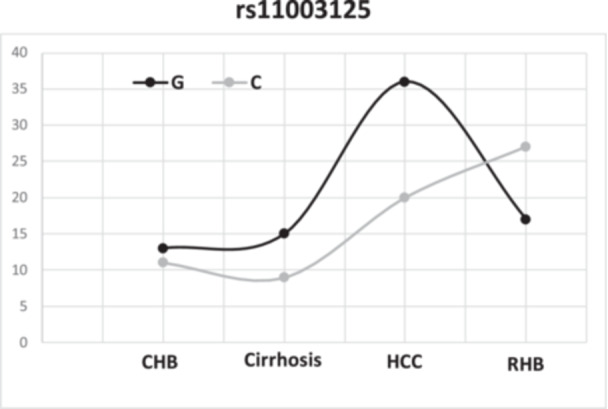
Distribution of rs11003125 alleles across HBV clinical stages. In the chronic, cirrhosis, and carcinoma group, the G allele was the most common, with 54.2% (13/24), 62.5% (15/24), and 64.3% (36/56), respectively, unlike in the resolved hepatitis group, where the C allele was dominant avec 61.4% (27/44).

Genotyping of MBL2 promoter variants revealed a predominance of the rs11003125 GC heterozygous genotype (77%, 57/74), followed by GG (16.2%, 12/74) and CC (6.7%, 5/74). Notably, the CC genotype was observed exclusively in individuals with resolved infection. The G allele was slightly more frequent overall (54.7%, 81/148) compared to the C allele (45.3%, 67/148), although allele distribution varied across clinical groups (Figure 1).

**Figure 2:**
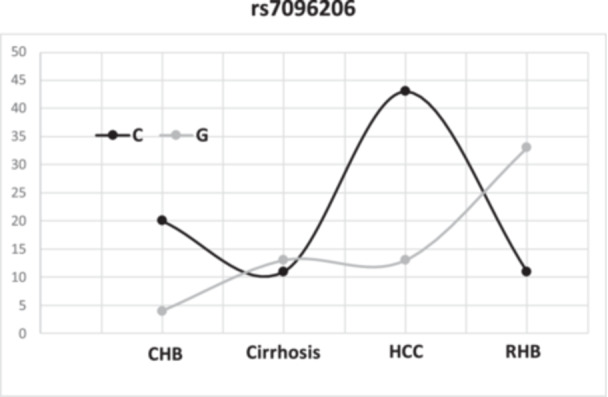
Distribution of rs7096206 alleles across HBV clinical stages. In the chronic cases and carcinomas group, the C allele was the most common, with 83.3% (20/24) and 76.8% (43/56) respectively, compared to 45.8% (11/24) and 25% (11/44) in the cirrhosis and resolved hepatitis group, where the G allele was dominant (54.2%: 13/24 and 75%: 33/44, respectively).

A study of the rs7096206 polymorphism revealed a frequency of 43.2% (32/74) for the wild‐type CC genotype and 28.4% (21/74) for the heterozygous CG and homozygous GG each. The “C” allele was the most represented at 57.4% (85/148), compared with 42.5% (63/148) for the “G” allele (Figure 2).

Also in the study population, genotype distribution differed between clinical subgroups.

## 4 Association Between MBL2 Gene Polymorphisms and Course of Infection

### 4.1 MBL2 Gene Polymorphism Frequencies in Chronic Hepatitis, Cirrhosis, HCC, and Resolved Hepatitis Groups

The genotypes and allelic frequencies of the two polymorphisms (rs7096206 and rs11003125) between patients with chronic hepatitis, cirrhosis, HCC and cases of resolved hepatitis are shown in Table 2.

**Table 2:** Comparative table of genotypic and allelic frequencies between CHB, cirrhosis, HCC, and RHB.

			HCC	RHB	OR (95% CI)	Fisher's exact test *p*
			*N* = 28 (%)	*N* = 22 (%)		
rs11003125	Genotypes	GG	8 (28.6)	0	Reference	
		GC	20 (71.4)	17 (77.3)	NA	0.01
		CC	0	5 (22.7)	NA	*p* < 0.001
	Alleles	G	36 (64.3)	17 (38.6)	Reference	
		C	20 (45.7)	27 (61.4)	0.3 (0.1–0.7)	0.01
rs7096206	Genotypes	CC	15 (53.6)	4 (18.2)	Reference	
		CG	13 (46.4)	3 (13.6)	1.1 (0.2–6.1)	*p* > 0.99
		GG	0	15 (68.2)	NA	*p* < 0.001
	Alleles	C	43 (76.8)	11 (25)	Reference	
		G	13 (23.2)	33 (75)	0.1 (0.04–0.25)	*p* < 0.001

			Cirrhosis	RHB	OR (95% CI)	Fisher's exact test *p*
			*N* = 12 (%)	*N* = 22 (%)		
rs11003125	Genotypes	GG	3 (25)	0	Reference	
		GC	9 (75)	17 (77.3)	NA	0.06
		CC	0	5 (22.7)	NA	0.01
	Alleles	G	15 (62.5)	17 (38.6)	Reference	
		C	9 (37.5)	27 (61.4)	0.3 (0.13–1.05)	0.07
rs7096206	Genotypes	CC	4 (33.3)	4 (18.2)	Reference	
		CG	3 (25)	3 (13.6)	1 (0.12–8.3)	*p* > 0.99
		GG	5 (41.7)	15 (68.2)	0.33 (0.05–1.85)	0.37
	Alleles	C	11 (45.8)	11 (25)	Reference	
		G	13 (54.2)	33 (75)	0.39 (0.13–1.13)	0.10

			CHB	RHB	OR (95% CI)	Fisher's exact test *p*
			*N* = 12 (%)	*N* = 22 (%)		
rs11003125	Genotypes	GG	1 (8.3)	0	Reference	
		GC	11 (91.7)	17 (77.3)	NA	0.41
		CC	0	5 (22.7)	NA	0.10
	Alleles	G	13 (54.2)	17 (38.6)	Reference	
		C	11 (45.8)	27 (61.4)	0.5 (0.19–1.45)	0.30
rs7096206	Genotypes	CC	9 (75)	4 (18.2)	Reference	
		CG	2 (16.7)	3 (13.6)	0.29 (0.03–2.52)	0.32
		GG	1 (8.3)	15 (68.2)	0.02 (0.002–0.3)	*p* < 0.001
	Alleles	C	20 (83.3)	11 (25)	Reference	
		G	4 (16.7)	33 (75)	0.06 (0.01–0.2)	*p* < 0.001

Abbreviation: NA = not applicable.

The rs11003125 and rs7096206 polymorphisms appear to be linked to the pathogenesis of HBV infection. Indeed, significant differences in genotypic and allelic frequencies were found for these two SNPs.

As shown in Table 2, the rs7096206 polymorphism was associated with protection against the progression of infection to the chronic phase. Indeed, when we compare the effects of the rs7096206 mutation between the chronic and resolved hepatitis groups, we find that the homozygous GG genotype and G allele were associated with “resolution of infection” (OR = 0.02; CI = 0.0002–0.3; *p* < 0.001 and OR = 0.06; CI = 0.01–0.2; *p* < 0.001, respectively, for the GG genotype and G allele).

Similarly, we found that the G allele of rs7096206 and the C allele of rs11003125 were correlated with a reduced risk of progression to HCC (OR = 0.1; CI = 0.04–0.25; *p* < 0.001 and OR = 0.3; CI = 0.1–0.7; *p* = 0.01, respectively for the G and C alleles).

This suggests that the presence of the mutation at the time of infection could promote viral elimination and therefore the resolution of the infection.

The homozygous CC mutated rs11003125 genotype also showed an association with the development of liver cirrhosis (*p* = 0.01).

### 4.2 Frequencies of MBL2 Gene Polymorphisms in Chronic Hepatitis, Cirrhosis, and HCC Groups

Comparison of frequencies between chronic hepatitis and HCC showed no statistical difference. Indeed, there was no significant association between these two polymorphisms and the progression from chronic hepatitis to HCC (*p* > 0.05) (Table 3).

**Table 3:** Comparative table of genotypic and allelic frequencies between CHB, cirrhosis, and HCC.

			CHB	Cirrhosis	OR (95% CI)	Fisher's exact test *p*
			*N* = 12 (%)	*N* = 12 (%)		
rs11003125	Genotypes	GG	1 (8.3)	3 (25)	Reference	
		GC	11 (91.7)	9 (75)	0.27 (0.02–3.09)	0.59
		CC	0	0	NA	NA
	Alleles	G	13 (54.2)	15 (62.5)	Reference	
		C	11 (45.8)	9 (37.5)	0.7 (0.2–2.2)	0.77
rs7096206	Genotypes	CC	9 (75)	4 (33.3)	Reference	
		CG	2 (16.7)	3 (25)	3.37 (0.39–28.74)	0.32
		GG	1 (8.3)	5 (41.7)	11.25 (0.97–130.2)	0.06
	Alleles	C	20 (83.3)	11 (45.8)	Reference	
		G	4 (16.7)	13 (54.2)	5.9 (1.5–22.5)	0.01

			CHB	HCC	OR (95% CI)	Fisher's exact test *p*
			*N* = 12 (%)	*N* = 28 (%)		
rs11003125	Genotypes	GG	1 (8.3)	8 (28.6)	Reference	
		GC	11 (91.7)	20 (71.4)	0.22 (0.02–2.06)	0.23
		CC	0	0	NA	NA
	Alleles	G	13 (54.2)	36 (64.3)	Reference	
		C	11 (45.8)	20 (45.7)	0.65 (0.24–1.73)	0.45
rs7096206	Genotypes	CC	9 (75)	15 (53.6)	Reference	
		CG	2 (16.7)	13 (46.4)	3.9 (0.7–21.4)	0.15
		GG	1 (8.3)	0	NA	0.40
	Alleles	C	20 (83.3)	43 (76.8)	Reference	
		G	4 (16.7)	13 (23.2)	1.5 (0.43–5.2)	0.76

			Cirrhosis	HCC	OR (95% CI)	Fisher's exact test *p*
			*N* = 12 (%)	*N* = 28 (%)		
rs11003125	Genotypes	GG	3 (25)	8 (28.6)	Reference	
		GC	9 (75)	20 (71.4)	0.8 (0.17–3.89)	*p* > 0.99
		CC	0	0	NA	NA
	Alleles	G	15 (62.5)	36 (64.3)	Reference	
		C	9 (37.5)	20 (45.7)	0.9 (0.34–2.49)	*p* > 0.99
rs7096206	Genotypes	CC	4 (33.3)	15 (53.6)	Reference	
		CG	3 (25)	13 (46.4)	1.15 (0.21–6.14)	*p* > 0.99
		GG	5 (41.7)	0	NA	0.003
	Alleles	C	11 (45.8)	43 (76.8)	Reference	
		G	13 (54.2)	13 (23.2)	0.25 (0.09–0.7)	0.009

Abbreviation: NA = not applicable.

However, the rs7096206 polymorphism was associated with a predisposition to cirrhosis. Indeed, we found that carriage of the rs7096206 G allele was associated with an elevated risk of developing severe forms (OR = 5.9; CI = 1.5–22.5; *p* = 0.01) (Table 3).

The rs11003125 polymorphism of the MBL2 gene revealed no significant association as to its involvement in the progression of chronic hepatitis to cirrhosis and cirrhosis to HCC (*p* > 0.05).

Our study showed that progression from cirrhosis to HCC was influenced by the G allele of rs7096206, which could confer protection (OR = 0.25; CI = 0.09–0.7; *p* = 0.009).

Homozygous GG mutated genotype of rs7096206 was also associated with the progression from cirrhosis to HCC (*p* = 0.003).

## 5 Discussion

In this study, we provide evidence that promoter polymorphisms in the MBL2 gene are associated with distinct clinical trajectories of HBV infection in a Burkinabe cohort. By integrating genotype distributions across disease stages, our findings support a context‐dependent role for MBL2 in modulating antiviral immunity, inflammatory injury, and disease progression.

The MBL protein is important during HBV infection. It may contribute to direct viral elimination, reduction of hepatic inflammatory damage through neutralization of immune complexes and downregulation of pro‐inflammatory cytokines [19, 20].

Hardy–Weinberg equilibrium tests were not performed in a local reference population due to the lack of a representative genetic database for Burkina Faso. However, Hardy–Weinberg equilibrium was calculated for our study population, but it was not in equilibrium due to small sample sizes.

The results showed that the heterozygous genotype of rs11003125 was the most prevalent in the population and, at the same time, in the different clinical groups (CHB, HCC, RHB, and cirrhosis). However, no association between the progression of chronic infection to cirrhosis or HCC was found for this polymorphism. Nevertheless, the heterozygous GC genotype (HCC vs. RHB) and homozygous mutated CC genotype (cirrhosis vs. RHB and HCC vs. RHB) were associated with the progression of infection. We also noted that the presence of the C allele of rs11003125 could reduce the risk of progression of the infection to HCC. Indeed, the C allele of rs11003125 has been associated with high serum MBL concentrations in studies of patients with dilated cardiomyopathy and type 2 diabetes. In these studies, the C allele increased the transcriptional activity of the MBL2 gene promoter, unlike the G allele [21, 22].

High expression of the MBL protein could contribute to improved viral clearance, as MBL can act as an opsonin and bind to HBV via specific receptors (mannose‐rich oligosaccharide), thereby promoting neutralization and elimination of the virus [23].

MBL could also indirectly influence the immune response by activating dendritic cells. This could strengthen the surveillance of precancerous cells and limit their expansion [24].

This improved response to viral infection would reduce chronic inflammation, thereby causing little liver damage, which would help reduce the risk of tumor transformation of hepatocytes and severe forms.

In the study of rs7096206 polymorphisms, the results showed that they were associated with disease progression. In other words, carriers of the G allele may have a very high predisposition to liver cirrhosis in the event of chronic infection. This trend was also found in a study conducted on patients of Chinese origin [25]. A study on the association between the MBL2 gene and chronic obstructive pulmonary disease revealed that the G allele may cause a deficiency in the MBL protein [26]. Our results are consistent with the hypothesis that low levels of MBL protein resulting from this mutation may be associated with the severity of infection [6]. Indeed, the G allele of rs7096206 has been correlated with a decrease in MBL levels [27]. MBL facilitates the phagocytosis of dead or infected cells. A deficiency in MBL leads to the accumulation of cellular debris in the liver, which exacerbates the inflammatory response [19]. In the absence of MBL, certain proinflammatory molecules such as TNF‐α and IL‐6 may be increased. This can lead to an unregulated response that contributes to damage to hepatocytes and promotes progression to cirrhosis [28]. This MBL deficiency could therefore reduce the immune response and viral clearance. This would result in persistent infection, prolonged liver inflammation, and the development of fibrosis and then cirrhosis.

Polymorphisms in the MBL2 gene could influence innate immunity and increase the risk of HCC [29, 30]. Indeed, MBL deficiency has been associated with impaired HBV clearance, persistent infection progressing to cirrhosis, and HCC [25]. MBL has the ability to activate complement on AgHbs‐MBL immune complexes via the lectin pathway, thereby contributing to viral clearance [31]. Low MBL levels associated with increased IL‐6 [15] may promote chronic inflammation and oxidative stress. IL‐6, in turn, can contribute to fibrogenesis by activating liver stellate cells and persistent HBV infection, leading to cirrhosis and HCC [32, 33].

Furthermore, our study also indicated that the G allele of rs7096206 may have a protective effect against chronic hepatitis, HCC, and the progression of cirrhosis to HCC.

If the G allele of rs7096206 of the MBL2 gene is associated with lower serum levels of MBL, and therefore with a weak immune response, how can we explain that it has a protective effect against the progression of infection in certain clinical groups?

CD8 T cells have been shown to be involved in immunity against HBV. A strong cytotoxic CD8 T cell response has been found in patients who are able to clear the virus and resolve the infection [34]. During the acute phase of infection, patients who are likely to eliminate the virus and recover from the infection develop a strong immune response by presenting the viral antigen to cytotoxic CD4 and CD8 T lymphocytes, which contribute to the lysis of infected hepatocytes, the blocking of viral replication, and the elimination of the virus via IFNγ, IFNα, and TNF‐α [35]. In fact, the MBL protein also contributes to the elimination of the virus via the regulation of TNF‐α [15]. This reduction in MBL2 gene activation could also limit the protumorigenic effects of chronic inflammation, explaining its possible protective role against the development of HCC in a cirrhotic context. A high level of MBL can promote virus clearance, resulting in an inflammatory response, which could prove harmful to the host. A low level (via the G allele) may not eliminate the virus as effectively, but it limits liver damage, hence the protective effect observed.

### 5.1 Limitations of the Study

This study has certain limitations. First, the relatively small sample size may limit the generalizability of the results to the entire study population. Genotyping was performed on all available samples. Only samples that were positive by real‐time PCR (well‐amplified and confirmed) were included in the statistical analyses. Internal controls were not systematically included due to logistical constraints. Despite these limitations, this research provides useful exploratory findings and serves as a foundation for future studies that can draw on larger samples and greater resources.

## 6 Conclusion

The rs11003125 and rs7096206 polymorphisms in the promoter region of the MBL2 gene appear to play a decisive role in the progression of HBV infection. The C allele of rs11003125, associated with high levels of serum MBL, may promote better viral clearance, thereby limiting chronic inflammation and progression to HCC. Conversely, the G allele of rs7096206, which decreases MBL expression, is linked to an increased risk of progression from CHB to cirrhosis, probably due to a weakened immune response.

These results confirm the involvement of the MBL2 gene in the innate immune response against HBV, illustrate the complexity of the immune role of MBL in liver diseases, and highlight the interest of these polymorphisms as potential genetic markers of susceptibility or protection in HBV infection. Further studies, incorporating gene interactions, are needed to refine their usefulness as prognostic tools or therapeutic targets.

## Author Contributions

Lassina Traore, Minane Nafissa Triande, Teega‐Wendé Clarisse Ouedraogo, Florencia Wendkuuni Djigma, and Jacques Simpore conceived and designed the study. Lassina Traore, Teega‐Wendé Clarisse Ouedraogo, Minane Nafissa Triande, Sidnooma Véronique Zongo, Sanata Nadine Kiemde, and Tegwindé Rébéca Compaore performed sampling and laboratory analyses. Lassina Traore, Teega‐Wendé Clarisse Ouedraogo, Minane Nafissa Triande, and Sanata Nadine Kiemde conducted statistical analyses and interpreted the data. Lassina Traore, Minane Nafissa Triande, Tegwindé Rébéca Compaore, Augustin Tozoula Bambara, Roger Arsène Sombie, and Patoinewendé Denise Ilboudo drafted the manuscript. Sidnooma Véronique Zongo, Mousso Savadogo, Bolni Marius Nagalo, Patoinewendé Denise Ilboudo, Tegwindé Rébéca Compaore, Albert Théophane Yonli, Augustin Tozoula Bambara, Roger Arsène Sombie, Florencia Wendkuuni Djigma, and Jacques Simpore critically revised the manuscript for important intellectual content. Lassina Traore, Bolni Marius Nagalo, Albert Théophane Yonli, Florencia Wendkuuni Djigma, and Jacques Simpore provided administrative, technical and material support. Bolni Marius Nagalo, Albert Théophane Yonli, Florencia Wendkuuni Djigma, and Jacques Simpore supervised the study. All authors read and approved the final manuscript and agreed on the order of authorship.

## Disclosure

The lead author Dr. Lassina Traore, PhD, Assistant Professor of Biochemistry, Molecular Biology, and Microbiology, affirms that this manuscript is an honest, accurate, and transparent account of the study being reported; that no important aspects of the study have been omitted; and that any discrepancies from the study as planned (and, if relevant, registered) have been explained.

## Ethics Statement

The study was approved by the Ethics Committee for Health Research (Reference No. 2022‐02‐027).

## Consent

Written informed consent was obtained from all participants. Data confidentiality was ensured through storage on a password‐protected computer.

## Conflicts of Interest

The authors declare no conflicts of interest.

## Data Availability

The data sets generated and/or analyzed during this study are available from the corresponding author upon reasonable request. Dr. Lassina Traore, PhD, Assistant Professor of Biochemistry, Molecular Biology, and Microbiology, had full access to all data in this study and takes full responsibility for the integrity of the data and the accuracy of the data analysis.
